# Calibration Method of Measuring Heads for Testing Residual Stresses in Sheet Metal Using the Barkhausen Method

**DOI:** 10.3390/ma17184584

**Published:** 2024-09-18

**Authors:** Tomasz Garstka, Piotr Szota, Sebastian Mróz, Grzegorz Stradomski, Jakub Gróbarczyk, Radosław Gryczkowski

**Affiliations:** 1 Faculty of Production Engineering and Materials Technology, Czestochowa University of Technology, 42-201 Częstochowa, Poland; tomasz.garstka@pcz.pl (T.G.); sebastian.mroz@pcz.pl (S.M.); grzegorz.stradomski@pcz.pl (G.S.); 2 Serwistal Sp. z.o.o., 2A Dojazdowa Str., 19-300 Ełk, Poland; j.grobarczyk@serwistal.pl (J.G.); r.gryczkowski@serwistal.pl (R.G.)

**Keywords:** non-destructive testing, residual stresses, FEM, Barkhausen effect

## Abstract

Among non-destructive testing methods, a group dedicated to the assessment of the state of residual stresses can be distinguished. The method of measuring residual stresses using the Barkhausen noise method has many advantages, as evidenced by the number of publications. The residual stresses in metal products are important for the further processing of such metal, such as laser cutting or bending. The results presented in this work are of an experimental nature, and the presented method of calibration of measuring heads shows how various research techniques can be used to correlate results. The research was carried out for structural steel due to the market share of this type of steel. The method can be used to measure the residual stresses in ferromagnetic metal products in order to assess their directions and quantify them. A prerequisite for the use of this measurement method is that the amplitude and geometry of the Barkhausen noise are adequately correlated to the specific values of the state of stress depending on the tested steel grade or other metals. In this study, a method for calibrating measuring sensors for the residual stress measurements is presented, as developed by the authors. The method involved conducting bending tests in both numerical modeling and experimental tests. During the bending tests, changes in the magnetic field (Barkhausen noise waveform) were recorded, taking into account the state of elastic stresses. Correlating the results of the numerical calculations and Barkhausen noise measurements made it possible to determine the quantitative values of the residual stresses in the steel sheets. Thanks to the method used, very accurate measurement is possible, and the obtained results are repeatable.

## 1. Introduction

In the various industries involved in the manufacture and processing of steel products, the residual stresses present in sections and flat products cause considerable difficulties both directly at the production stage, when partial relaxation of the residual stresses is the cause of their deformation, and during their further processing [1,2,3].

In the case of semi-finished flat-rolled products, such as steel sheets or plates, during such technological operations as cutting, bending, stamping or welding, the residual stresses (mainly of the first and second order) accumulated in their volume are released, resulting in a change in geometrical dimensions and the deformation of the intended shape of the finished product [4,5]. The process of residual stress release can also occur during the use of finished products under the influence of a variety of factors, such as temperature, vibration, or external loads [6].

One of the causes of unfavorable internal stresses in metal products is the non-uniform material properties that are caused by preceding manufacturing processes. In particular, this is related to uneven cooling during the hot-rolling process or strong plastic strain [7,8,9,10]. Depending on the extent of the effect, residual stresses can be divided into the following types [11]:

- Type I stresses (σ′—macro stresses), occurring throughout the continuum of the test object. They are caused by the macro-influence of a number of external factors, such as the variation in the internal structure caused by the machining and uneven cooling at different depths. These stresses mainly cause changes in workpiece dimensions and fractures.
- Type II stresses (σ″—micro stresses), occurring in the area of several adjacent grains and their boundaries, are caused by changes in the orientation of the grains with respect to each other and by the difference in specific volume between them.
- Type III stresses (σ‴—sub-micro stresses), affecting the region of several atomic distances and caused by numerous defects in the structure of the crystalline lattice after processing (vacancies, interstitial atoms, dislocations, lattice cracks, and delaminations).

In sheet metal production, knowledge of the distributions and values of the residual stresses [12,13,14,15] allows for (corrective) measures to be taken at individual stages of their manufacture, e.g., during straightening; aimed at obtaining the intended stress characteristics, reducing their absolute values, or obtaining more harmonized surface distributions.

As assessment tools, the various methods of measuring residual stresses can be broadly divided into two groups: destructive and non-destructive methods. The use of destructive testing techniques, such as sectioning, removing layers of material, or drilling, only allows for the evaluation of certain manufactured components in a limited area, resulting in permanent damage or the destruction of the test object [16,17,18]. Such testing, despite the analytically valuable methodology resulting from the direct link between the revealed strain and stress, is usually very time-consuming and expensive. The use of non-destructive methods in industrial practice is of particular importance. Measuring and testing methods are based on a non-invasive approach [19], enabling rapid inspection of entire batches of manufactured components, and they are based on measuring parameters of physical phenomena, e. g., magnetic, the course of which depends on the properties of the material being analyzed [20]. In the steel industry, in addition to previously used measurement techniques, such as X-ray [21,22] and ultrasound [23,24], the Barkhausen effect method [25,26,27,28,29] is becoming increasingly popular for analyzing the level of residual stresses. This technique makes it possible not only to assess the distribution and value of the intrinsic stresses but also, to some extent, to analyze the microstructure of the steel, its chemical composition or other parameters, such as microhardness [30,31,32]. In terms of measurement depth, this method allows values of up to several hundred micrometers to be reached, i.e., a greater depth than with the X-ray method and with better selectivity than the ultrasonic method [33]. Another important advantage of this method is the measurement speed, which is at the level of just a few seconds in automated systems.

Direct comparison of numerical values of stress measurement results at given points using the Barkhausen method with other different methods is difficult and generally incorrect, because each of the popular methods actually measures stresses of different types from different areas. However, a good agreement is observed, when carefully analyzed, between the results of measurements using the Barkhausen method and strain methods or ultrasonic tests [14].

The technique is named after Heinrich Barkhausen, who, in 1919, discovered the fundamental physical phenomenon underlying this method. This phenomenon (the so-called Barkhausen effect) occurs in ferromagnetic materials during their magnetization process. Under the influence of an alternating magnetic field generated, for example, by a yoke-type electromagnet applied to the material, a cyclic remodeling of the domain structure takes place. This process involves a change in orientation through rotation of the magnetic domains and a stochastic, step-wise increase in volume through shifting of their boundaries (Bloch walls), known as Barkhausen jumps [34,35]. This part of the process, associated with overcoming energy barriers appearing, for example, at defects in the microstructure that limit free reorganization, results in an increase in the material’s magnetization from the external magnetic field not occurring smoothly, but rather in a stepwise manner. As a result, each single pulse of magnetization increase associated with a domain wall shift will generate in the detection coil near the material surface [36,37]. During a single magnetization cycle, after filtering out the low-frequency component of the magnetizing current and its harmonics from the detection coil signal, a characteristic voltage noise spectrum containing between a few hundred hertz and a few hundred kilohertz is obtained, called *magnetic Barkhausen noise (MBN)* [38]. In the generalized case, the course of the step-wise shifts in the domain walls is determined by a number of different factors, such as the previously mentioned chemical composition, the state of internal stresses, and the microstructure or level of magnetization of the test material, among others. The parameters of the detected Barkhausen noise depend on the above factors [39]. *MBN* can be electrically characterized using parameters, such as amplitude *A_MBN_*, the rms value *RMS_MBN_*, the number of induced Barkhausen pulses of a given amplitude per unit time (usually one magnetization cycle) *N_C_*, or the frequency spectrum. In addition, the shape of the *MBN* envelope can be described using geometrical parameters and time dependencies related to the position of the peaks on the envelope relative to the zero crossing point of the magnetic field strength waveform.

The measurement of residual stresses by this method—treating other factors related to the microstructure or magnetization as invariant—is based on the comparison of one of the *MBN* parameters, often the *RMS* value or the number of Barkhausen jumps that are measured in the test material, with the following calibration characteristic (1):

(1) $$X_{MBN}=f(\sigma )$$

Equation (1) describes the dependence of the selected *X_MSB_* parameter as a function of stress ó and is usually established in a reverse bend or tensile/compressive test, introducing a known state of stress into the calibration sample [40].

Over a large range, the increase in the *RMS* value of *MBN* as a function of changing stress (in the sense from compressive *−*σ to tensile *+*σ with a zero crossing) is monotonic, and the shape of (1) is sigmoidal. The greatest dynamics of change in *MBN* parameters are observed in the range of small and medium stress values with respect to yield strength. After certain characteristic values of tensile and compressive stresses are exceeded, curvature of the characteristics and a transition to the so-called state of saturation are observed. An increase in stress in this area no longer results in an increase in *MBN* parameters, and sometimes even a reversal of the monotonicity of the characteristic is observed, as a result of a change in the sign of the magnetostrictive coefficient for high stress values. The level of stress at which the breakdown of the characteristic and the transition to the saturation region occurs is usually defined as a percentage of the yield stress *YS* or the offset yield strength *YS*_0.2_.

The values of these stresses depend not only on the properties of the material itself, but also on the design of the measuring equipment and the measuring conditions. The value of the saturation stress, so far, determines the range of testing possibilities with this method.

Augustyniak, in his paper [40], determined the range of stresses for steel of the St3S grade, causing measurable increases in the *Nc* number of Barkhausen jumps in the range from −50% to 100% of the yield stress. Lindgren [41], for the corrosion-resistant Avesta 2205 duplex steel (with a 47% austenite content), estimated these limits at −200 MPa for compressive stresses and 250 MPa for tensile stresses. Silva, in his paper [42], determined calibration curves for the *RMS* of *MBN* in ASTM A 515 steel with a yield strength of 310 MPa. The saturation stress values he estimated were, on average, 50% for tensile stresses and 30% for compressive stresses of the *YS*_0.2_ value. In the abovementioned paper [42] and articles [43,44,45], it was observed that there was a pronounced anisotropy in the magnetomechanical properties of the test material in the direction in line with and transverse to the rolling direction. In unloaded samples, the *RMS* level of Barkhausen noise was significantly different in samples cut along the rolling direction, which was also reflected in other parameters. For this reason, residual stresses must be accurately determined on calibration samples made of a material with the same microstructure and texture, but also with an analogous strain history as the test object.

An important aspect of the calibration process is the selection of a suitable function approximating the so-called calibration curve or scaling function (2), which is the inverse of Equation (1).

(2) $$\sigma =f(X_{MBN})$$

The simplest mathematical models of the dependence of the intensity variation of a selected *MBN* parameter as a function of stress can even be assumed to be linear [26]. In order to provide a better representation, other, more complex methods are necessary, most often using polynomials or exponential functions. Tonshoff, in his paper [46], presented a way to determine stress by means of an equation that relates the weighted sum of three *MBN* parameters: the amplitude, the time shift of the peak in the envelope, and the number of Barkhausen pulses. A mathematical model of the calibration function (based on the *MBN RMS* value) in the form of a third-degree polynomial and a linear term taking into account the effect of temperature was used by Venginovich [47], achieving, with their use, a high accuracy of the description of the experimentally determined curve. Augustyniak [40], for approximation of the calibration function of strain ε, used an equation which was the sum of parameterized exponential functions and a linear function, for which the argument was also the rms value of *MBN*.

This paper proposes a Barkhausen noise calibration method based on numerical modeling by means of the finite element method of the state of stress in a test sample subjected to a reverse bend test under real experimental conditions. On the other hand, advanced mathematical models based on the use of hyperbolic trigonometric functions were used to model the relevant calibration functions. By using this method, it is possible to quantify the values of residual stresses with a high degree of accuracy.

The aim of the study was to develop *MBN* characteristics to accurately measure the values of residual stresses and their directions in a 3 mm-thick sheet of the S355J2 steel grade (EN 10025-2/DIN17100).

## 2. Research Methods

As part of the work, a method of calibrating heads using the Barkhausen effect to measure residual stresses was developed. The method involves examining sheet metal bending in numerical modeling and experimental studies, and then correlating these results. The research sought the relationship between the state of deformation and stress in the elastic state (numerical modeling) and the noise characteristics during demagnetization. Measuring the above parameters for the appropriate roller displacement allowed for the correlation of results. The research was divided into two stages. In the first stage, numerical calculations were performed using computer software based on FEM. In the second stage, the characteristics of Barkhausen noise in the actual state of deformation for S355J2 steel were determined using the designed device for sample deformation. Based on the results of these studies, it was possible to calibrate the heads. Combining theoretical calculations with experimental results using the developed calibration method allowed for the quantitative determination of residual stress levels with high accuracy.

### 2.1. Computer Simulations

Computer simulations of the steel samples bending were carried out using an elastic-plastic model of material deformation, in which the mechanical state of the deformed material is described using the following Norton–Hoff law (3) [48,49,50]:

(3) $$S_{ij}=2K(T,\dot{\bar{\varepsilon }},\bar{\varepsilon }){\left(\sqrt{3}\dot{\bar{\varepsilon }}\right)}^{m-1}{\dot{\varepsilon }}_{ij}\;[\mathrm{MPa}]$$

where *S_ij_*—stress tensor deviator, $\dot{\bar{\varepsilon }}$—strain rate intensity, ${\dot{\varepsilon }}_{ij}$—strain rate tensor, $\bar{\varepsilon }$—strain intensity, *T*—temperature, *K*—stress-dependent consistency *σ*, and *m*—coefficient describing the hot deformation of the metal (0 < *m* < 1).

The applied model was verified by comparing the tensile force values from the numerical modeling and static tensile test of the S355J2 steel samples (reverse method). The tensile force deviation value was 2.1%, while the length deviation after stretching was 0.4%.

Steel grade S355J2 was used for this study as it is one of the most commonly used grades for structural component production (Table 1). Hot-rolled sheet with a thickness of 3 mm and a width of 1500 mm was supplied in coils. Using the MTS E45.305 testing machine (max. force of 300 kN), static tensile tests were carried out to determine the yield stress curves of the steel grade tested. Static tensile tests were carried out using dumbbell-shaped samples in accordance with the current standards for static tensile testing [51].

**Figure 1:**
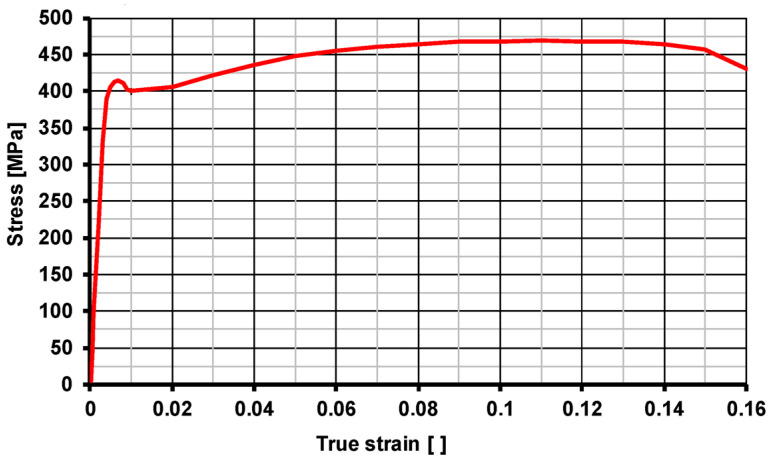
Plastic flow curve of S355J2 steel.

A model of the properties of steel grade S355J2 was introduced to the model described by Equation (3) (Figure 1). To make the calculations more accurate, the curve was introduced in a tabular form, a graphical representation of which is shown in Figure 1. The use of tabulated values allows for the variability in the plasticity curve to be taken into account, especially in the areas of the plastic limit, which is much more difficult in the case of functions. In addition, the models developed accounted for the physical properties of the steel grade used (Table 1), as well as the yield strength YS and Young’s modulus (Table 2).

**Figure 2:**
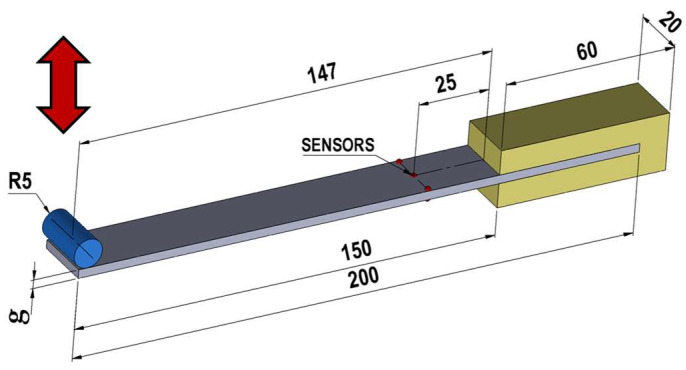
Geometrical model of the bending used in the numerical calculations, g = 3 mm.

The computer simulation also requires the following to be entered: friction coefficient μ—0.15; sheet temperature—20 °C; Poisson coefficient—0.3; roller displacement speed—0.5 mm/s; specific heat—480 J/(kg·K); density—7850 kg/m^3^; conductivity—29.9 W/(m·K). A schematic of the geometrical model used in the numerical calculations is shown in Figure 2.

The samples were rigidly fixed on one side, while on the other side, the roller was bending the sample (see arrow in Figure 2). The sample was assumed to bend up and down by 7 mm in each direction. Stress values were recorded in 1 mm increments. The stresses were recorded at a distance of 25 mm from the point of fixing the sample in the clamps (see sensors in Figure 2). For this purpose, the sample being deformed was introduced with 3 sensors on the top surface and 3 on the bottom surface. The sensors were mounted 0.1 mm below the surface of the sample to eliminate the possibility of erroneous readings of stress values as a result of numerical errors. The values obtained for the individual sensors were averaged. The value of the maximum bending of the sample (±7 mm), which was adopted for the numerical calculations, corresponded to the elastic range of deformation for the steel tested.

### 2.2. Experimental Testing Methodology

**Figure 3:**
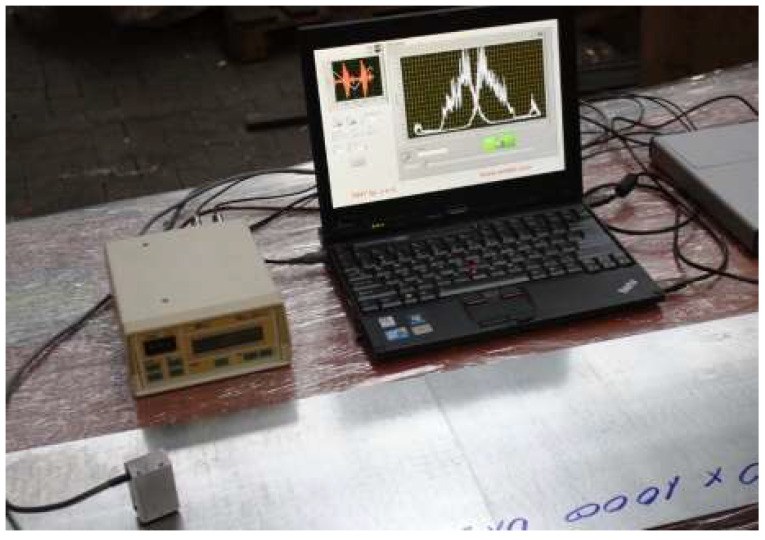
Example view of the measuring system with the MEB-2C gauge [52] used to test residual stresses using the Barkhausen method.

Barkhausen noise measurements were carried out using a MEB-2C digital Barkhausen effect meter (Mag-Lab s.c., Gdańsk, Poland) [52], with a unidirectional measuring head and a data acquisition and recording system. An image of the measurement system is shown in Figure 3.

This meter allows for the determination of its energy parameter based on the measured *MBN*, i.e., the *RMS* value of the *MBN*. This value, as an unmeasured value, in digital form together with information about the magnetization conditions, is sent to a portable computer recording the data. In addition, the Barkhausen noise from the output of the measuring track is acquired by the measurement card and also saved as a sample.

**Figure 4:**
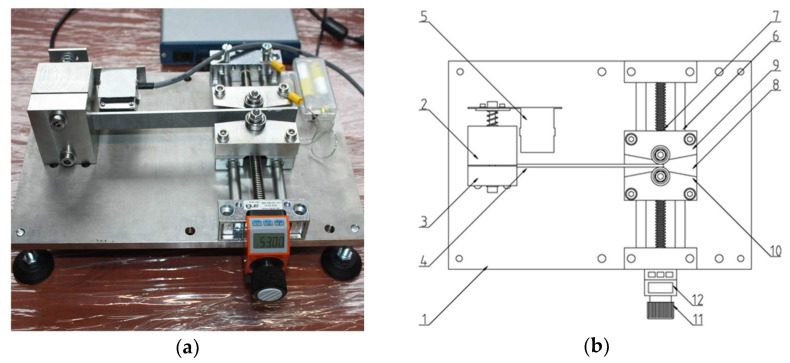
Calibration sample deformation instrument (**a**), components (**b**): 1—base, 2, 3—calibration sample holder, 4—calibration sample, 5—calibration head holder, 6—guide rail, 7—lead screw, 8— carriage, 9, 10—bearing mandrel supports 11—knob, and 12—position indicator.

The initial test plan was to calibrate using a testing machine, consisting of tension and compression of the samples. However, due to the initial bending of some of the test samples, fitting them in the jaws of the testing machine would have caused them to straighten, introducing additional stresses to the calibration. For this reason, the tests were carried out in the designed instrument shown in Figure 4.

Figure 4a shows a view of the instrument and Figure 4b identifies its main components. Its construction rests on a base (1), which is a 10 mm-thick plate that provides adequate rigidity and stability to the structure. Attached to the base is one part of the holder (2) with a cut-out for mounting the calibration sample (4), to which a second, complementary part of the holder (3) and the calibration head mounting system (5) are attached.

An assembly for the deformation of the calibration sample is mounted on the base. It consists of a linear guide profile rail (6), along which a carriage with polymer slide bearings (8), driven by a lead screw (7). Supports with bearing mandrels (9) and (10) are bolted to the carriage, directly deforming the sample during its movement. The position of the carriage is changed using a knob (11), and an electronic indicator (12) is used for precise position measurement. The instrument is additionally equipped with an electrical circuit to signal contact between the mandrel and the sample. All components of the instrument are made of austenitic steel, or other non-magnetic materials, to reduce the possibility of interference due to Barkhausen noise excitation.

**Figure 5:**
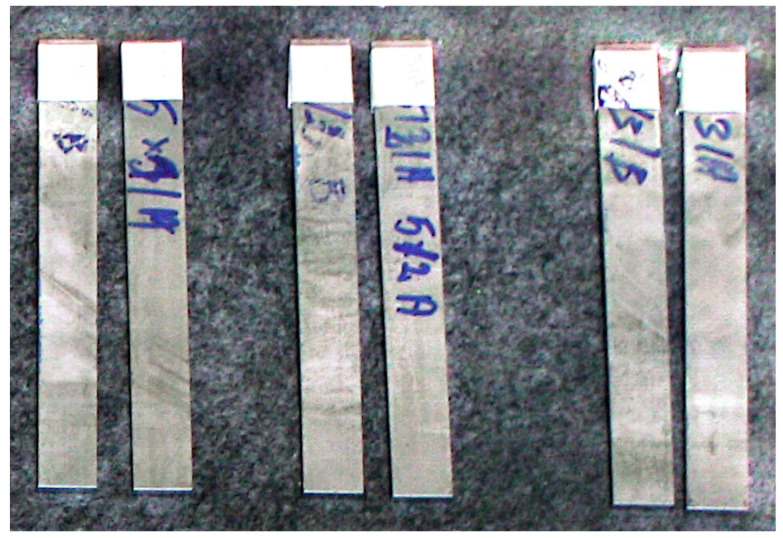
View of calibration samples.

**Figure 6:**
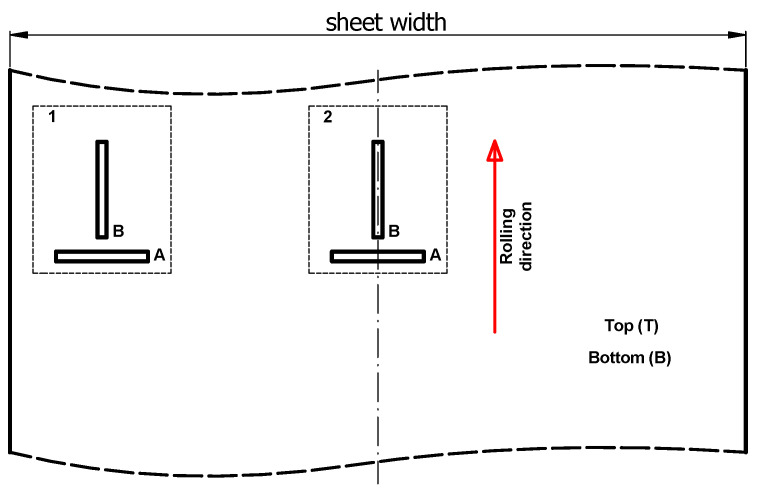
Locations of samples for the calibration process and their designations (1—sample in the sheet axis, 2—samples at the sheet edge, A—sample cut transversely to the rolling direction, B—sample cut along the rolling direction.

In order to quantify the residual stress level, it is necessary to prepare calibration characteristics of the Barkhausen noise intensity as a function of the stress *MBN = f (σ)*. Calibration was carried out on samples that were deformed by introducing a known state of stress. In order to achieve high calibration accuracy, the samples must have similar microstructure and mechanical properties as the tested sheet. For this reason, samples were taken from representative locations across the width of the tested sheets, i.e., from the center and edges. Such calibration must, moreover, be carried out for both the rolling and transverse directions as well as the top and bottom sides of the sheet. For this reason, rectangular samples of 200 mm × 20 mm were taken for calibration. A view of examples of calibration samples taken is shown in Figure 5. Figure 6 shows the method of taking samples from a 3000 mm × 1500 mm sheet of metal.

## 3. Results of the Conducted Tests

**Figure 7:**
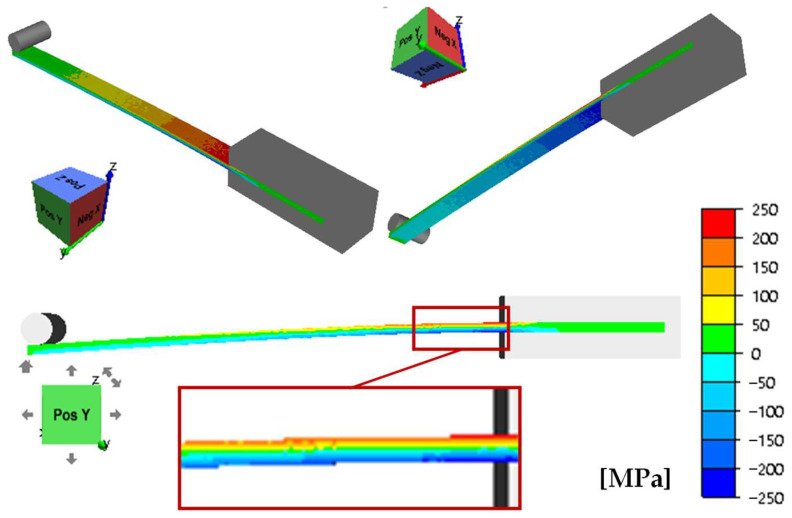
Stress distribution σ_x_ in a sample made of S355J2-grade steel (max. bend of 7 mm).

In the theoretical part of the study, the distribution of stress tensor component σ_x_, effective stress σ, and strain ε from a minimum value of 1 mm to a maximum value of 7 mm was determined. The analysis for the individual components was performed for the elastic range (Figure 7).

**Figure 8:**
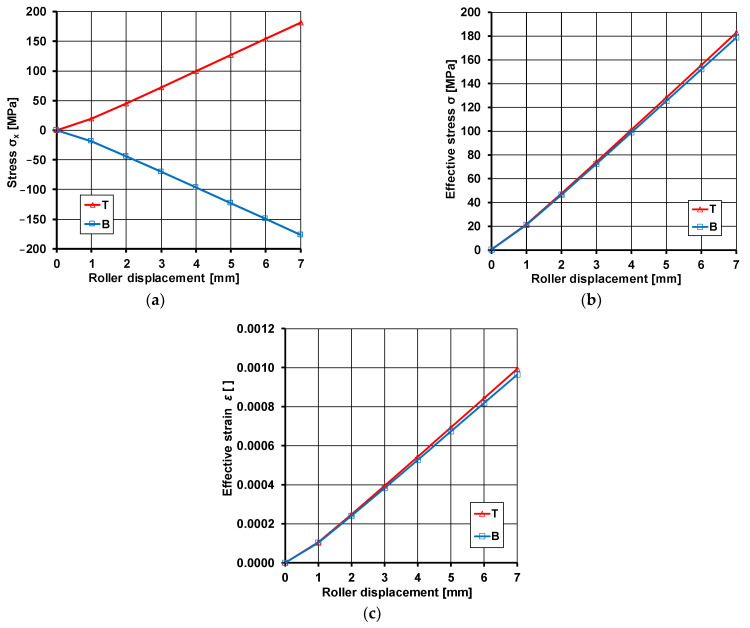
Results of numerical modeling of bending of S355J2 sheet samples: (**a**) stress *σ_x_*, (**b**) effective stress *σ*, and (**c**) effective strain *ε*.

The data presented in Figure 7 show that the maximum value of tensile stress on the top surface of the sample or compressive stress on its bottom surface for maximum bending reaches a value of 178.8 MPa, which is less than the yield strength. As expected, when the sample is bent downwards, there are tensile stresses on the top surface of the sample and compressive stresses on the bottom surface of the sample. Figure 8a shows the course of change in stress *σ_x_*, effective stress *σ* (Figure 8b), and effective strain ε (Figure 8c) from a minimum value of 1 mm to a maximum value of 7 mm. The values obtained are the averages from the three sensors shown in Figure 2.

From Figure 8a, it can be seen that the course of change in the obtained values of the *σ_x_* component is the same as far as the absolute value is concerned, and differs only in sign, which is consistent with the bending direction. The course of change is linear (elastic range of deformation). However, as mentioned above, the maximum values are lower than the yield strength. An analysis of the data presented (Figure 8b) leads to the conclusion that the values obtained are the same for both the top and bottom surfaces. Slight differences were noted at higher values of sample deflection. Again, the maximum value of the effective strain is lower than the yield strength. The course of the effective strain changes shown in Figure 8c is identical to the course of the effective stress. The obtained effective strain value is less than 0.2% of the permanent deformation, meaning that the sample was in the elastic range during bending (Table 3).

**Figure 9:**
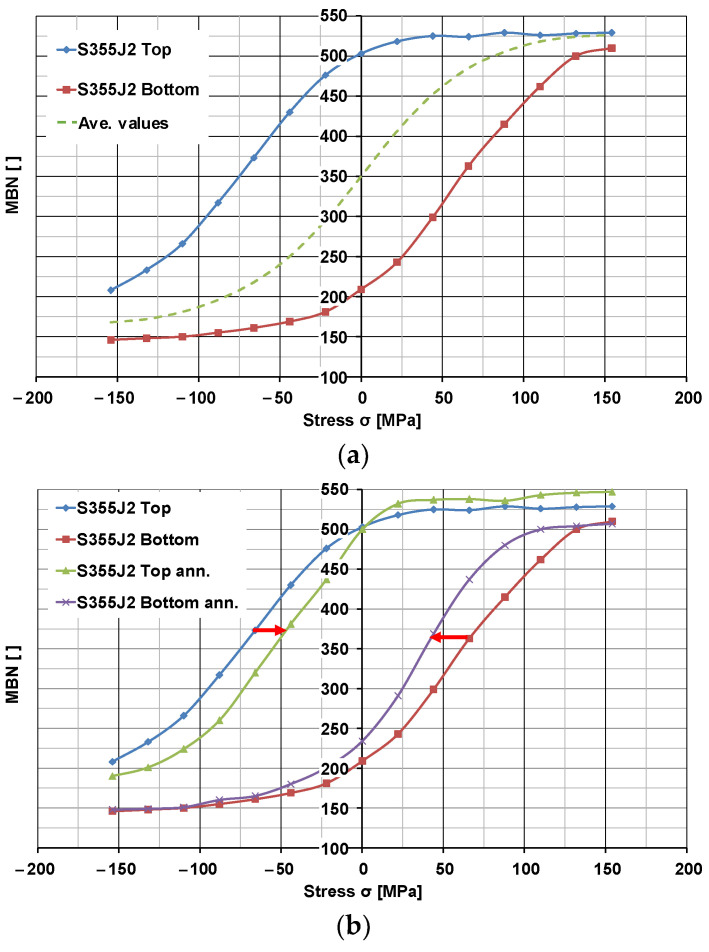
Experimental results for S355J2 steel samples: (**a**) calibration curve and (**b**) shift in calibration curves in the sample with residual stresses before and after stress-relief annealing.

In order to correctly calibrate the *MBN* characteristics and to be able to compare the stress values obtained in numerical modeling and experiment, it was necessary to carry out stress-relief annealing. The annealing was carried out in a temperature range that did not alter the microstructure of the test samples (annealing for 2 h at 300 °C). As a result of the temperature treatment, the stresses resulting from rolling and coiling were removed. The samples prepared in this way were used to carry out the calibration by employing the instrument developed for the controlled and precise application of compressive and tensile stresses on the surface of the samples and a Barkhausen noise measurement device. The analysis resulted in the development of characteristic curves approximating the sigmoidal dependence of *MBN* intensity as a function of the applied stress σ varying in both value and sign, *MBN = f (σ)*. Figure 9a shows an example of a typical calibration curve obtained for S355J2-grade steel. The calibration was carried out in the safe range of deformations, with the course of the calibration curve being monitored during the calibration, so as not to exceed the yield strength.

Figure 9b shows a very striking example of calibration curves in a sample, where residual stresses were maintained at +80 MPa on the top surface and at −70 MPa on the bottom surface. This state of stress, superimposed on the applied stresses, obviously shifted the resulting curves horizontally relative to the calibration curve characteristic of this steel grade, indicated by the dashed line. This resulted in a rapid transition of such curves into their plateau region.

Calibration procedures were carried out for samples that had undergone stress-relief annealing. Analysis of the calibration curves for annealed samples revealed that, for some samples with relatively high residual stresses, it was not possible to fully relax them for the applied annealing parameters that do not affect the microstructure. A representative example of such a case is shown in Figure 9b.

The data shown in Figure 9 contain a flat part at the ends of the ranges of the calibration curves, where calibration was no longer effective, so the curves were supplemented iteratively with *MSB* values (1) being the sum/difference in the result of the last measurement and an expression described by a power function with fractional exponent *k*, for which the argument was the absolute value from the difference in the last two measurements.

(4) $$MSB_{n+1}=MSB_{n}\pm {\left|MSB_{n}-MSB_{n-1}\right|}^{k}$$

Based on the research results in Table 4 and Table 5, replacement curves were drawn up as a result of averaging the waveforms. Such a procedure, thanks to a statistical approach, makes it possible to obtain calibration curves that are representative of the analyzed steel grade and to determine their characteristic parameters.

**Figure 10:**
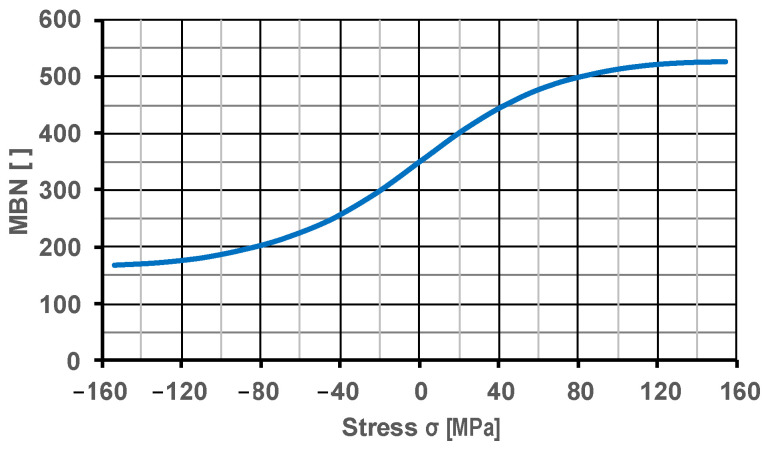
Course of the calibration curve for S355J2 steel.

Based on the replacement calibration curves (Figure 10), universal computational mathematical models of the calibration function *σ = f (MBN)* were then developed, which can be used to directly determine the values of the residual stresses from the measurement results. While the mathematical description of the shape of the experimental calibration curves does not pose any major problems when, e.g., the logistic or Boltzmann function are used, it is a challenge to describe the calibration functions derived from them, which have two vertical asymptotes. The use of a polynomial to describe the calibration function requires the use of a fifth-degree polynomial. Such a description has unfavorable interpolation characteristics and associated low utility outside the area of interpolated data, and, in addition, its practical use requires a very precise implementation in the computational model of the determined coefficients. For this reason, it was decided to look for new forms of description based on hyperbolic functions, which approximate the shape of the calibration functions.

Initially using the hyperbolic sine function (5) as a basis function, satisfactory results were obtained for the modeling of the calibration functions, where the average relative error of approximation was at the level of several percent, but when relating the individual absolute differences to the yield stress value *Y_S_*, this value drops to only 2% [53]. The coefficients of Function (5) are given in Table 6. Function (5) is as follows:

(5) $$\sigma =a_{3}\cdot \sinh\;\left(a_{2}\cdot \left(MBN+a_{1}\right)\right)+a_{0}$$

where *a*_0_*, a*_1_, *a*_2_, *a*_3,_—coefficients, and *σ*—stress [MPa].

In order to increase the accuracy of the determination of the values of residual stresses in the sheet in the further course of the study, calibration functions were determined for the individual regions of the sheet, i.e., the center of the sheet and its edges, and for the top and bottom surfaces of the sheet, respectively. 

The diagrams use the designations of samples taken from selected areas of the sheets (Figure 6), for which calibrations were carried out (first digit—edge/center, first letter—cutting direction, second letter—top/bottom).

The shapes of the calibration curves obtained from the wide range of calibration procedures carried out for representative areas of the sheets were harmonized with respect to the previously determined replacement curves. In order to increase the accuracy of the calibration functions, especially in their bending areas, model (5) was evaluated to form (6), where the argument of the basis function *sinh* is a hyperbolic tangent operating on a modified value of the measured *MBN*. The values of the coefficients are given in Table 7. Equation (6) is as follows:

(6) $$\sigma =a_{4}\cdot sinh\left(a_{3}\cdot tanh\left(a_{2}\right)\cdot \left(MBN+a_{1}\right)\right)+a_{0}$$

where *a*_0_, *a*_1_, *a*_2_, *a*_3_, *a*_4_,—coefficients, and *σ*—stress [MPa].

The orders of magnitude of the individual parameters and their approximate values were tentatively determined empirically. However, the exact values of the coefficients of Equation (6) for each calibration function were determined using the non-linear Marquardt–Levenberg least squares algorithm [54,55,56].

Although the set of calibration function values for a given strain (deflection) of the sample could have been calculated from analytical relations, the values from numerical modeling of the deformation of the calibration samples were adopted for calibration as these are more accurate, taking into account the actual mechanical properties of the steel grade tested. The mathematical model of the approximating function developed, based on hyperbolic functions, has very good mapping accuracy, allowing the particularly sensitive areas of the transition of calibration curves to saturation to be modeled perfectly. It should be noted that the form of the mathematical model adopted, while having very good accuracy, does not have the interpolation features characteristic of a polynomial description. 

**Figure 11:**
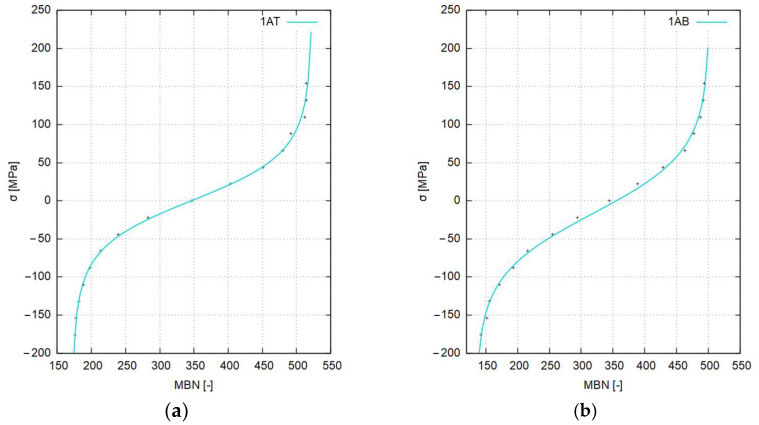

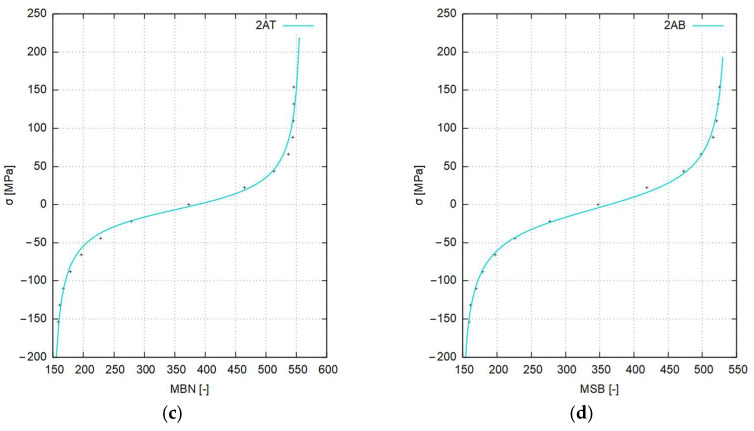
Traverse calibration functions for S355J2-grade steel: (**a**) band edge—top of the sheet, (**b**) band edge—bottom of the sheet, (**c**) band center—top of the sheet, and (**d**) band sheet—bottom of the sheet.

**Figure 12:**
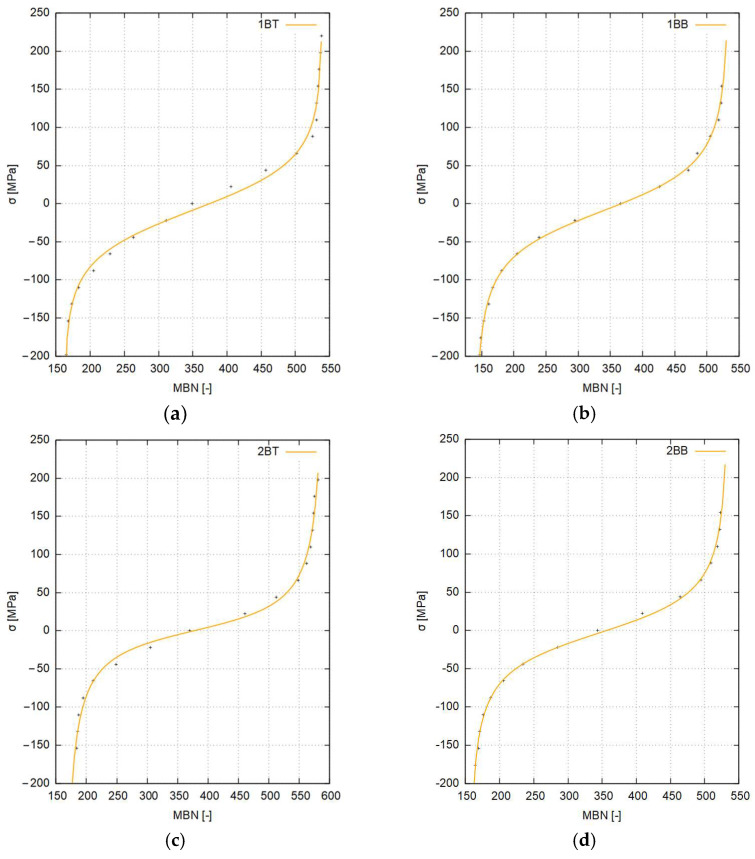
Longitudinal calibration functions for S355J2-grade steel: (**a**) band edge—top of the sheet, (**b**) band edge—bottom of the sheet, (**c**) band center—top of the sheet, and (**d**) band sheet—bottom of the sheet.

The shapes of the calibration functions and the experimental data (+marked) are presented as graphs in Figure 11 and Figure 12.

All curves described by functions have a similar course passing through the 0 MPa point at the MBN value of about 350. However, they differ in the inclination of the straight line sections and the values in the saturation areas at high values of compressive and tensile stresses, respectively.

## 4. Conclusions

The paper presents the methodology of calibration stress measurements using the Barkhausen method. On the basis of obtained results, it was possible to make the following statements:

1. The use of multi-method technique for calibrating stress measurements using the Barkhausen method employing numerical modeling as well as an experimental reverse bend test of sheet metal with a specially designed measuring instrument give very accurate results.

2. On the basis of the obtained results of reverse numerical calculations of sheet bending for S355J2-grade steel, the definition of stress changes depending on the bending of the sample was possible to determine.

3. The obtained results of the theoretical calculations were used during the calibration, and on the basis of the obtained stress values, it was possible to correlate the characteristics of the magnetization noise with the values of stresses in the sheets.

4. The results obtained from modeling the shape of the calibration functions, using the sine and hyperbolic tangent, are satisfactory, with an error of less than 10%.

5. The error is caused by the assumption is accounted for, and a method of controlling the parameterization process of the model makes it possible for it to be functional also outside the area of the experimental learning data and not to have strict vertical asymptotes. The coefficient of determination *R*^2^ also reaches a very high value, which indicates a good choice of the form of the calibration function.

6. The use of the developed calibration functions in the measurements allows the measurements of the residual stresses of S355J2 steel to be carried out with high accuracy.

## Figures and Tables

**Table 1:** Chemical composition of S355J2 steel grade, PN-EN 10052, [%].

	C	Mn	Si	P	S	Cr	Mo	V	Nb	Ti	Al	Cu
PN-EN S355J2	0.20	1.60	0.55	Max 0.025	Max 0.025	-	-	-	-	-	-	Max 0.55
Tested S355J2	1.185	1.5	0.3	0.005	0.005	-	-	-	-	-	0.003	0.1

**Table 2:** Mechanical properties of the tested steel grade (hot-rolled).

Steel Grade	Yield Strength YS [MPa]	Strength Limit UTS [MPa]	Young’s Modulus E [GPa]	Max. Absolute Error Δ [MPa]	Max. Relative Error δ [%]
S355J2	410	468	181	±4.28	0.91

**Table 3:** Summary results of numerical calculations.

Steel	Measured Parameter	Roller Displacement Value [mm]						
		1	2	3	4	5	6	7
S355J2	Effective stress σ [MPa]	21.2	46.5	72.4	98.9	125.4	152.1	178.8
	Effective strain ε [ - ]	0.000104	0.000245	0.000389	0.000536	0.000683	0.000830	0.000979

**Table 4:** Results of Barkhausen noise intensity measurements on calibration samples.

Sample Deflection, [mm]	S355J2 in the As-Delivered Condition							
	Edge of Sheet 1				Center of Sheet 2			
	Transverse A		Longitudinal B		Transverse A		Longitudinal B	
	Top	Bottom	Top	Bottom	Top	Bottom	Top	Bottom
−7	178	140	208	146	165	155	179	164
−6	179	142	233	148	167	157	185	166
−5	181	144	266	150	173	163	197	167
−4	185	149	317	155	183	173	219	169
−3	193	167	373	161	199	189	259	177
−2	203	195	430	169	231	216	329	197
−1	220	226	476	181	275	260	413	225
0	251	272	503	209	368	333	483	283
1	300	313	518	243	450	404	533	345
2	360	365	525	299	497	456	553	413
3	412	408	524	363	518	481	566	466
4	450	441	529	415	526	501	567	488
5	471	466	526	462	526	509	568	502
6	476	471	528	469	526	512	569	506
7	478	473	529	471	526	514	570	508

**Table 5:** Results of Barkhausen noise intensity measurements in annealed calibration samples.

Sample Deflection, [mm]	S355J2 in Annealed Condition							
	Edge of Sheet 1				Center of Sheet 2			
	Transverse A		Longitudinal B		Transverse A		Longitudinal B	
	Top	Bottom	Top	Bottom	Top	Bottom	Top	Bottom
−7	173	160	201	148	153	162	189	172
−6	173	163	224	149	155	165	192	175
−5	179	175	260	151	161	175	203	183
−4	183	191	315	160	173	185	239	197
−3	192	205	325	165	193	205	281	215
−2	207	237	381	180	225	235	327	243
−1	227	275	437	201	281	293	438	287
0	266	322	500	234	377	363	491	341
1	333	369	532	291	479	433	542	404
2	396	417	537	369	528	490	557	462
3	452	460	538	437	555	514	570	501
4	489	482	536	480	561	530	575	516
5	508	507	543	500	563	532	578	532
6	512	512	546	504	565	533	580	536
7	514	514	547	507	566	535	581	538

**Table 6:** Coefficients of Equation (5) for the plate tested.

Steel Grade	*a*_0_	*a*_1_	*a*_2_	*a*_3_	R^2^	δ_%_ δ_%YE_ [%]
S355J2	−2.88626	−347.141	0.0163897	15.4037	0.9966	9.7 1.5

**Table 7:** Values of calibration function coefficients for S355J2 steel.

No.	Sample *	*a*_0_	*a*_1_	*a*_2_	*a*_3_	*a*_4_	R^2^	δ_%_ [%]
1	1BT	148.733	0.442600	0.00529000	−350.60	−8.4100	0.9964	10.1
2	1BB	78.716	0.828892	0.00504245	−337.17	−9.88456	0.9991	4.2
3	2BT	25.312	1.652280	0.00461565	−378.52	0.1846	0.9959	10.6
4	2BB	55.340	1.014560	0.00524665	−345.48	−3.4401	0.9986	5.0
5	1AT	109.001	0.589351	0.00566458	−347.285	0.38637	0,9977	4,9
6	1AB	339.924	0.240834	0.00544643	−318.057	−16.8533	0.9986	7.3
7	2AT	26.870	1.410490	0.00480442	−354.319	−6.11260	0.9910	11.0
8	2AB	43.287	1.160367	0.00508767	−341.671	−5.80557	0.9980	6.7

* marking according to Figure 6.

## Data Availability

Data are contained within the article.
